# Development of a Weight-Drop Impact Testing Method for Dental Applications

**DOI:** 10.3390/polym12122803

**Published:** 2020-11-26

**Authors:** Satoru Watanabe, Yoshiki Ishida, Daisuke Miura, Taira Miyasaka, Akikazu Shinya

**Affiliations:** 1 Department of Dental Materials Science, School of Life Dentistry at Tokyo, The Nippon Dental University, 1-9-20 Fujimi, Chiyoda-ku, Tokyo 102-8159, Japan; s-watanabe_d2117004@tky.ndu.ac.jp (S.W.); yishida@tky.ndu.ac.jp (Y.I.); daisuke@tky.ndu.ac.jp (D.M.); miyasaka@tky.ndu.ac.jp (T.M.); 2 Department of Life Science Dentistry, School of Life Dentistry at Tokyo, The Nippon Dental University, 1-9-20 Fujimi, Chiyoda-ku, Tokyo 102-8159, Japan; 3 Department of Prosthetic Dentistry and Biomaterials Science, Institute of Dentistry, University of Turku, Lemminkaisenkatu 2, 20520 Turku, Finland

**Keywords:** CAD/CAM blocks, denture base materials, dynamic test method, impact strength, weight-drop impact test

## Abstract

For evaluating the impact strength of dental materials, the Izod test or Charpy test has been used, but specimen preparation for these tests is difficult due to the adjustment of a notch on them. By contrast, a weight-drop impact test does not require notched specimens. Therefore, it might be possible to measure the impact strength more accurately than conventional methods. This study aimed to establish appropriate conditions for applying the weight-drop impact test on small specimens of acrylic resin. To determine the most reliable impact fracture energy of acrylic resins, different diameters and thicknesses of PMMA resin specimens, diameters and weights of the striker, and diameters of the supporting jig were compared. For all specimen thicknesses, when the striker diameter was 6–10 mm, the impact fracture energy was constant when the inner diameter of the specimen-supporting jig was 8–10 mm. In addition, the measured E_50%_ value was mostly equal to the median value of the impact fracture energy. Thus, for the weight-drop impact test, this method was effective for material testing of small specimens, by clearly specifying the test conditions, such as the thickness of disc-shaped specimens, the diameter of the striker, and the inner diameter of the specimen-supporting jig.

## 1. Introduction

Numerous impact forces, including instantaneous occlusal pressure [1], falling of dentures [2], and occlusal tapping during sleep [3], exert a load on dental materials in daily life. Such impact forces sometimes cause fractures or cracks in dental prostheses. Dynamic test methods are used to assess the characteristics of dental materials to withstand these impacts. These tests differ from tests of static mechanical strength, such as tensile strength and hardness, and demonstrate the behavior characteristic of the materials [4].

Notably, due to the increasing demand for oral esthetics, tooth colored materials such as ceramic-based have been widely used for dental restoration [5,6]. However, there have been some reports that these materials have a higher failure rate due to their brittle property [7,8,9]. Thus, it is important to evaluate the impact strength of tooth colored materials for further development.

To evaluate the impact strength of dental materials, impact tests, such as the Izod test or Charpy test, have been used in dentistry [10,11,12,13]. However, adjustment of test fragments (e.g., notch) is difficult [14] in conventional impact testing methods. Moreover, as the measured values vary markedly [15], it is challenging to measure the impact strength of dental materials accurately using these methods. Furthermore, for esthetic restorations, computer-aided design and computer-aided manufacturing (CAD/CAM) systems are widely used for the production of dental prostheses, and the majority of materials for the CAD/CAM system are mainly available in discs and rectangular-shaped blocks [16]. It is impossible to obtain specimens for the conventional impact methods from materials for the CAD/CAM system because of their small size.

On the other hand, the impact testing method using a weight-drop is recommended for many polymers in the industrial field, and it has high reproducibility and reliability [17,18,19]. For plastic materials, the weight-drop impact testing method has been specified in the International Standards Organization (ISO) [20,21]. 

In dentistry, plastic materials have been widely used as denture base resins in daily clinical practice. Therefore, it is possible to apply the impact method to measure the impact strength of denture base resin by changing the specimen size. Furthermore, if it is possible to apply this test method for small-sized specimens, it may also be applicable to materials used for crown restorations, where there is a limit to the size of specimens. This would allow us to evaluate the impact strength of dental materials more accurately than by conventional methods and might facilitate further development of dental materials.

The ultimate goal of this project was to establish the application of the weight-drop impact test on small specimens of rigid dental materials. The specific aim of this study was to investigate the correct use of the weight-drop impact testing method by reducing the test fragment size to evaluate the impact strength. To this end, we prepared a prototype weight-drop impact testing machine for small specimens and investigated the details of the conditions of the test method, including the test fragment size.

## 2. Materials and Methods 

### 2.1. Experimental Design

**Figure 1:**
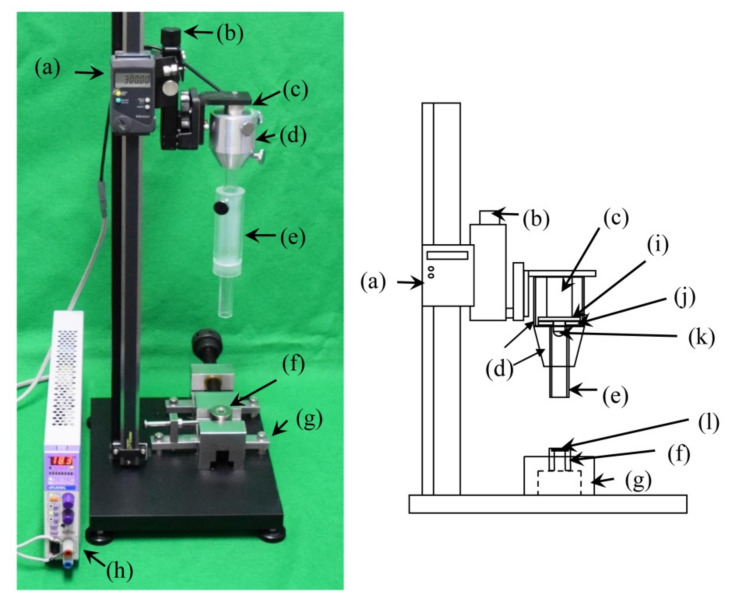
Prototype puncture impact test machine: (a) height measuring instrument, (b) micro-stage for fine height adjustment, (c) electromagnet, (d) guide shaft, (e) guide, (f) specimen-supporting jig, (g) vice grip, (h) power supply for electromagnet, (i) acrylic plate, (j) striker position-fixing plate, (k) striker, (l) specimen.

**Figure 2:**
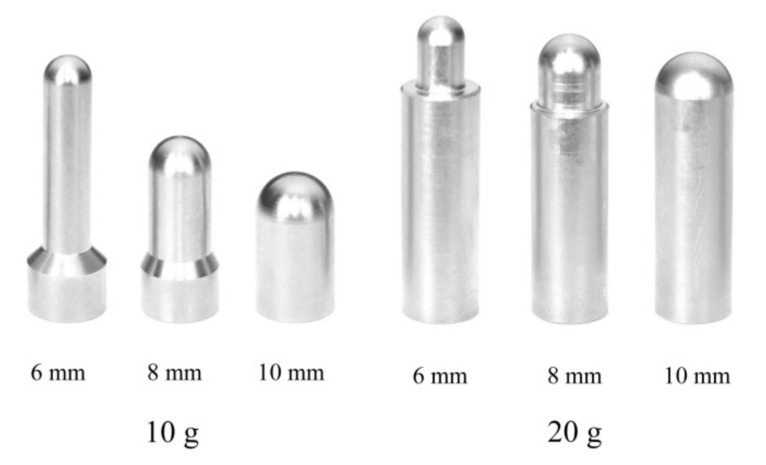
Striker.

**Figure 3:**
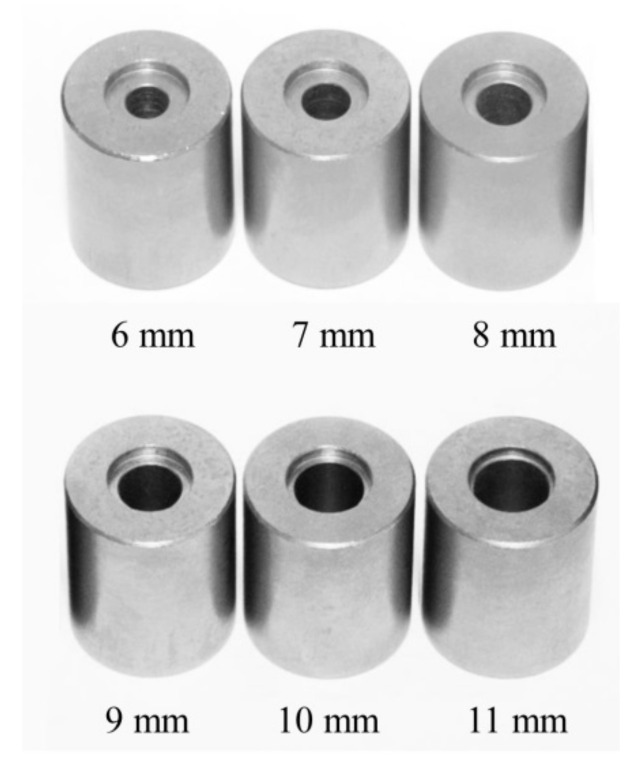
Specimen-supporting jigs.

To evaluate the testing conditions, a prototype weight-drop impact testing machine for small specimens (Figure 1) was constructed based on the standard weight-drop puncture impact testing method for plastic materials (ISO 6603-1:2000). First, a specimen diameter of 12 mm was selected in this experimental condition, because the average dental CAD/CAM block size is 14.5 mm wide, 18 mm high, and 14.5 mm deep. We selected three different specimen thicknesses (1.0, 1.5, 2.0 mm), based on the occlusal thickness of dental prosthetics made of rigid dental materials. For the purpose of scale adjustment in the decreased specimen diameter as compared to ISO, all additional parts, such as the strikers and specimen-supporting jigs were modified as follows. Three different striker tips with diameters of 6, 8, and 10 mm (Figure 2) were fabricated. In addition, the specimen-supporting jig (Figure 3), which has an outer diameter of 12 mm, but with six different inner diameters from 6 mm to 11 mm were fabricated. All these experimental conditions with different combinations of specimen thickness/striker diameter/supporting jig inner diameters were randomly compared to find suitable testing conditions for a small specimen made of rigid dental material.

### 2.2. Specimen Preparation

For specimen preparation, a polymethyl methacrylate (PMMA) resin plate (COMOGLASS, Kuraray, Tokyo, Japan), with 2000 mm length ×1000 mm width ×1.0, 1.5, and 2.0 mm thicknesses, was used as the experimental material. One-hundred-and-twenty disc-shaped specimens (diameter: 12 mm) were cut using a laser processing machine (GCC LaserPro Spirit GLS, GCC, New Taipei City, Taiwan) from each thickness of the acrylic resin plate. After specimens were fabricated, the thickness and diameter of all specimens were measured three times, and the mean errors were −0.03 to +0.04 mm, −0.02 to +0.03 mm, and 0.00 to +0.07 mm for 1, 1.5, and 2 mm thicknesses, respectively. The mean diameter error was −0.02 to + 0.04 mm. The specimen edge was visually confirmed to be a right angle, under a digital microscope (VHX-2000, KEYENCE, Osaka, Japan). The specimens were subjected to the weight-drop test after immersion in water at 37 °C for 24 h. 

### 2.3. Essential Components of Prototype Weight-Drop Impact Testing Machine

The prototype weight-drop impact testing machine was constructed following the standard weight-drop puncture impact testing method for plastic material (ISO 6603-1:2000). Figure 1 shows the prototype weight-drop impact testing machine. The prototype was designed as follows: The holding and releasing system for an energy carrier, such as a weighted striker and the striker position-fixing plate (diameter: 10 mm, Japan MECC, Tokyo, Japan, Figure 1j), was attached to the guide shaft (Japan MECC, Tokyo, Japan, Figure 1d) for a weighted striker installation tool to allow dropping of a striker in the vertical direction. This striker guide installation tool was attached to the sliding stand (maximum height: 600 mm, Microstand MK-2, SFC, Tokyo, Japan), keeping the striker position-fixing plate vertically, and an electromagnet (KE-2B, KANETEC, Nagano, Japan, Figure 1c) was connected to a power supply (ePL80WL, Fujitsu, Tokyo, Japan, Figure 1h) that was set in the guide shaft. To prevent the magnetization of the striker, a thin acrylic plate was set between the striker and the electromagnet. To measure the height of the fall, a height measuring instrument, with a micro adjustment (ABS Digimatic Length Measuring Unit SDV-60E, Mitutoyo, Kanagawa, Japan, Figure 1a) was connected to the sliding stand. In addition, to support the specimen during the weight-drop test, a specimen-supporting jig (height: 30 mm, outer diameter: 25 mm, Japan MECC, Tokyo, Japan, Figure 1f) was fixed onto the base by a vice grip (Japan MECC, Tokyo, Japan, Figure 1g). 

### 2.4. Size and Weight of Striker

To compare the different impact forces, columnar strikers (Japan MECC, Tokyo, Japan), attached to a carbon steel ball, with diameters of 6, 8, and 10 mm (Figure 2) were used. A striker weight of 10 g and 20 g was fabricated (Japan MECC, Tokyo, Japan). From the results of the preliminary experiment, 1 and 1.5 mm-thick specimens were fractured by using a 10 g striker. Therefore, the 10 g striker was used for all 1 and 1.5 mm-thick specimens. In the 2.0 mm-thick specimen, the 10 g striker was not fractured; thus, a 20 g striker was used for the 2.0 mm-thick specimen. The weight of the strikers was calculated as 10.0 ± 0.1 g and 20.0 ± 0.2 g, respectively. 

### 2.5. Supporting Jig

For the specimen-supporting jig, as shown in Figure 3, the outer diameter of the specimen-supporting part was fixed at 12 mm, and the inner diameter of the penetrating part was changed incrementally by 1 mm, from 6 to 11 mm, to prepare six different inner diameters (Figure 3). The outer/inner diameter ratio ranged from 0.5 (6/12) to 0.92 (11/12), respectively. 

### 2.6. Weight-Drop Impact Test 

The weight-drop impact test was performed following ISO standards (ISO 6603-1:2000) for assessing puncture impact behavior of rigid plastics. In the testing method, a specimen was first set onto the specimen-supporting jig, and the lower end of the striker was placed in contact with the upper surface of specimens to perform the zero setting on the height measurement instrument. Then, the holding and releasing system was pulled up and set at the specified height for the fall, and the power source was turned off to allow the striker to fall freely into the specimen. 

To determine the starting height, a pretest was performed using a 10 g striker with a supporting jig with an inner diameter of 6 mm for each specimen thickness and striker diameter combination, to determine the expected impact fracture energy. From the results of the pretest, the mean height causing fracture of the specimen and standard deviation were determined. The value calculated by subtracting the standard deviation from the minimum height causing destruction at each thickness was regarded as the starting height of the weight-drop test in this experiment. When the specimen was not destroyed at the starting height, the height was increased by 10 mm, and the test was repeated. The height was sequentially increased by 10 mm until the specimen fractured. This weight-drop test was continued until the specimen was fractured, for 20 specimens under each experimental condition (*n* = 20). 

The potential energy (E = mgH, m: mass of striker, g: gravitational acceleration) was calculated from the measured value of the height (H), which caused impact destruction of the specimens, and was regarded as the impact fracture energy. 

In addition, from the destructive test results of 20 specimens tested under the same test conditions, the 50% impact fracture energy (E_50%_) was calculated, using the following Equations (1)–(4) [20,21]:

E_50%_ = mgH_50_ = mg[H_a_ + ΔH(A/N − 1/2)] (1)

(2) $$N\;=\sum_{i=1}^{k}n_{i}$$

(3) $$A\;=\sum_{i=1}^{k}n_{i}z_{i}$$

z_i_ = H_i_ − H_a_/ΔH (4)

where m is the falling mass (kg), g is the acceleration of gravity (m/s^2^), H_a_ is the minimum height at the Kth height H_i_(I = 1 to K) (m), ΔH is the change in height (m), N is the number of fractures (calculated for N as N = N_x_ because N_x_ < N_o_); x: fractured; o: not fractured; n_i_: number of fractures at each height H_i_; z_i_: number of changes in height from H_a_.

### 2.7. Statistical Analysis 

The impact fracture energy was calculated from the height by changing the striker diameter (6, 8, and 10 mm) and inner diameter of the specimen-supporting jig (6, 7, 8, 9, 10, and 11 mm) for each specimen thickness (1, 1.5, and 2 mm), and this was regarded as the measured value of the specimen. 

Homoscedasticity of the measured values was analyzed using Bartlett’s test. As no homoscedasticity of variance was confirmed under any condition, the Kruskal–Wallis test was performed for each specimen thickness as a nonparametric statistical analysis. A highly significant difference was noted among the combinations of the striker diameter and inner diameter of the specimen-supporting jig at each specimen thickness (*p* < 0.01); therefore, all combinations were subjected to multiple comparisons using the Steel‒Dwass test. These statistical analyses were performed using R (www.r-project.org).

## 3. Results

**Figure 4:**
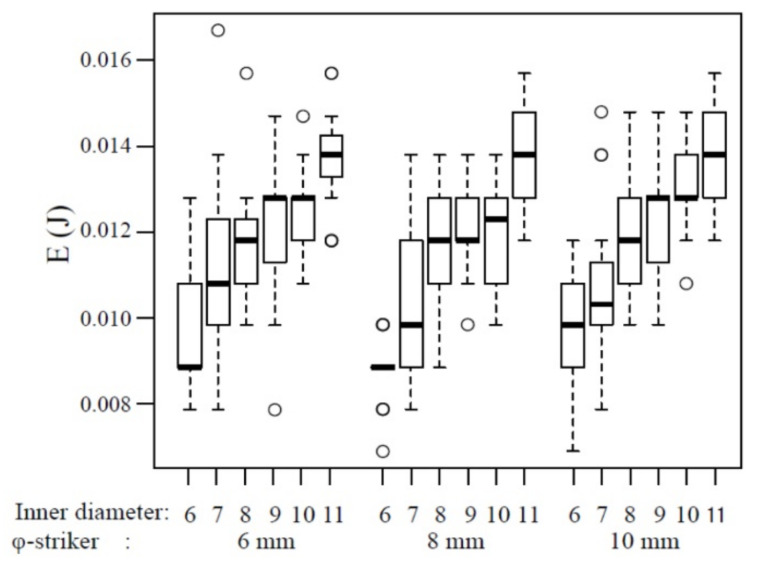
The impact fracture energy of 1 mm thickness. o: outliers.

The box-and-whisker plots of the impact fracture energy on 1 mm-thick specimens are shown in Figure 4. The horizontal line represents the median and the box the interquartile range (IQR). Each whisker is 1.5 times the IQR, and values that are outside this range are referred to as outliers and are denoted by a circle. In addition, the results of multiple comparisons are shown in Table 1. The median impact fracture energy increased slightly with the increase in the inner diameter at all striker diameters. However, no significant difference was noted at a 6‒8 mm diameter when the inner diameter of the specimen-supporting jig was 7‒10 mm (*p* > 0.05). When the striker diameter was 10 mm, a significant difference was observed between the specimen-supporting jig with 7 and 10 mm inner diameters (*p* < 0.05), but no significant differences were noted for the other combinations (*p* > 0.05). 

**Figure 5:**
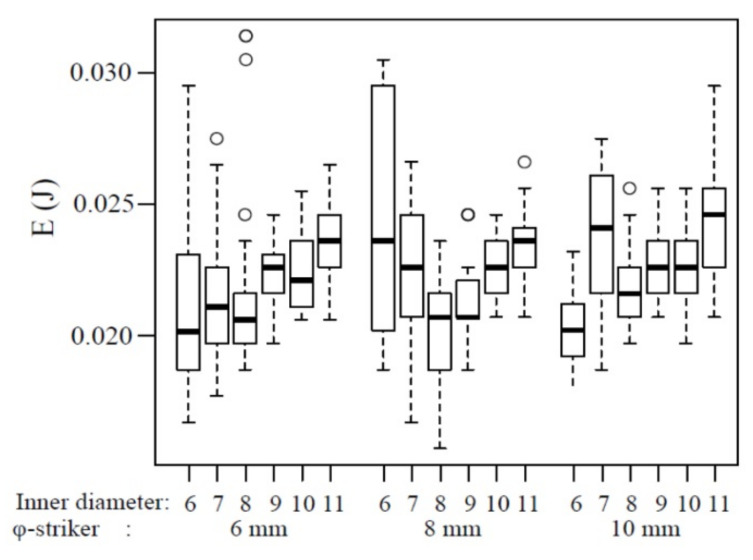
The impact fracture energy of 1.5 mm thickness. o: outliers.

The box-and-whisker plots of the impact fracture energy of 1.5 mm-thick specimens are shown in Figure 5. The horizontal line represents the median and the box the interquartile range (IQR). Each whisker is 1.5 times the IQR, and values that are outside this range are referred to as outliers and are denoted by a circle. In addition, the results of multiple comparisons are shown in Table 2. No change in the impact fracture energy was observed with the increase in the inner diameter of the specimen-supporting jig. Other than the significant (*p* < 0.05) and highly significant differences (*p* < 0.01) observed between 8 and 11 mm inner diameters of the specimen-supporting jig at 6 and 8 mm striker diameters, respectively, no significant differences were shown among the other combinations of the inner diameter (*p* > 0.05). When the striker diameter was 10 mm, a significant difference (*p* < 0.05) was observed between the specimen-supporting jig with 6 and 7 mm inner diameters, and a highly significant difference (*p* < 0.01) was observed between the 6 mm and the 9 mm, 10 mm, and 11 mm inner diameters. However, no significant difference was noted for any of the other combinations (*p* > 0.05).

**Figure 6:**
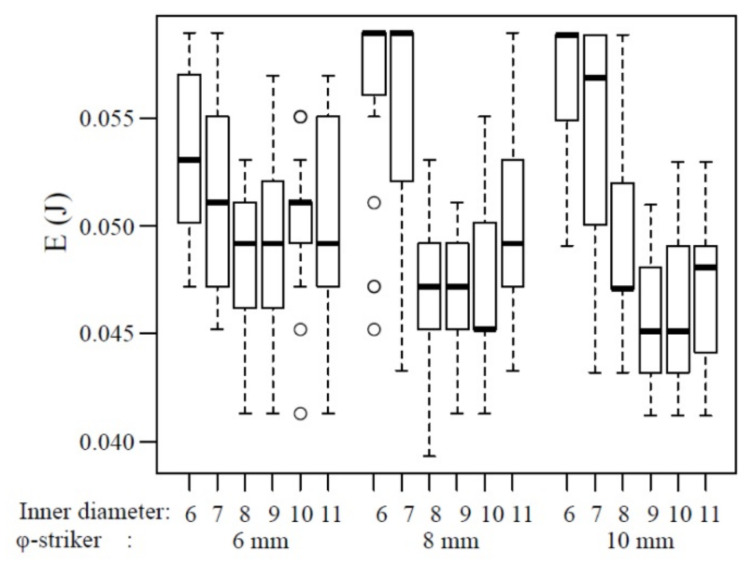
The impact fracture energy of 2 mm thickness. o: outliers.

The box-and-whisker plots of the impact fracture energy on 2 mm-thick specimens are shown in Figure 6. The horizontal line represents the median and the box the interquartile range (IQR). Each whisker is 1.5 times the IQR, and values that are outside this range are referred to as outliers and are denoted by a circle. In addition, the results of the Steel–Dwass multiple comparison are shown in Table 3. The impact fracture energy was slightly higher when the specimen-supporting jig had inner diameters of 6 and 7 mm than at the other inner diameters, at all striker diameters. No significant difference was observed for any combinations with a specimen-supporting jig of 7 mm or larger when the striker diameter was 6 mm (*p* > 0.05). When the striker diameter was 8 or 10 mm, no significant difference was observed for any combinations with a specimen-supporting jig with an inner diameter of 8 mm or larger (*p* > 0.05).

The E_50%_ was calculated under each condition: specimen thickness, striker diameter, and inner diameter of the specimen-supporting jig. The results are shown in Table 4, with the median measured values of impact fracture energy. When the specimen thickness was 1 mm, for all diameters of the striker, the E_50%_ slightly increased as the inner diameter of the specimen-supporting jig increased. When the specimen thickness was 1.5 mm or 2 mm, the E_50%_ was mostly similar for the 8 and 10 mm inner diameters of the specimen-supporting jig. 

## 4. Discussion

For impact testing of rigid dental materials, the Izod impact testing method [10,11,22] and the Charpy impact testing method [13,14,23] have been widely used. These methods make use of notched specimens. These notches can be made by adding the notch after specimen preparation, creating the notch during specimen preparation, or by cutting into the material surface with a blade [14]. However, these methods for preparing a notch are somewhat problematic. Adding the notch after specimen preparation might cause residual stresses within the specimens and cause hidden cracks. Creating the notch during preparation might cause a change in the radius of the notch tip, and the use of a blade might cause notches of variable depth, with or without associated cracks that may be undetected [14]. For these reasons, it is difficult to prepare specimens for accurate measurement of impact resistance. In addition, these conventional impact testing methods require a plate test specimen with a relatively large size. Owing to the recommendation for specimen size, it is challenging to apply these methods to measuring the impact fracture energy of rigid dental materials made of CAD/CAM blocks. Consequently, in this study, to investigate an impact testing method applicable to the size of dental CAD/CAM block materials, a prototype weight-drop impact testing machine for small specimens was prepared. For the weight-drop impact testing, a puncture impact testing method [20,21] is specified for industrial rigid plastics (e.g., acrylonitrile butadiene styrene resin, polyethylene resin, and polycarbonate resin [24]) in the ISO specifications (ISO6603-1:2000) [21]. However, the specimen size used in this method is too large to make disk-shaped specimens from rigid dental materials, and it was not suited to the objective of this study. Thus, the specimen size was reduced to a diameter of 12 mm and a specimen-supporting jig was prepared, and the relationship between the inner diameter of the specimen-supporting jig and the diameter of the striker that was able to punch out the specimen was set as a test condition. Changes in this relationship by changing the test conditions and the optimum condition of the weight-drop impact test were investigated. For the striker diameter, three conditions (6, 7, and 8 mm) were set, for which the inner diameter of the penetrating part of the specimen-supporting jig was changed incrementally by 1 mm, from 6 to 11 mm. The influence of specimen thickness (1, 1.5, or 2 mm) on the combinations of these experimental conditions was evaluated. For all specimen thicknesses, when the striker diameter was 6‒10 mm, the impact fracture energy was constant when the inner diameter of the specimen-supporting jig was 8‒10 mm. The measured E_50%_ value was mostly equal to the median value of the impact fracture energy. Thus, we showed that this method was effective for material testing of rigid dental materials by the weight-drop method.

For the 1 mm-thick specimens, a striker with a diameter of 6 mm penetrated a specimen-supporting jig with an inner diameter of 6 mm or larger. However, an inner diameter of 6 mm is the same as the diameter of the striker, suggesting that the fracture energy was the smallest due to the influence of the edges of the striker and specimen-supporting jig. Conversely, when the inner diameter of the specimen-supporting jig was 11 mm, the specimen lay on the jig in a 0.5 mm width, in a donut pattern, in which the distance between the central part of the specimen and the edge of the specimen-supporting jig was the largest. This may have been the reason for the largest apparent breaking energy. A significant difference was noted when the specimen-supporting jig had other inner diameters (7‒10 mm), and an almost identical impact fracture energy was acquired by setting the inner diameter of the specimen-supporting jig to 7‒10 mm when the striker diameter was 6 mm. Similarly, when the striker diameter was set at 8 mm, no significant difference was noted among the specimen-supporting jigs with 7‒10-mm inner diameters. However, based on Figure 4, the median was slightly lower at an inner diameter of 7 mm; thus, it may be better to consider that the impact fracture energy was almost identical when the inner diameter is 8–10 mm. When the striker diameter was set at 10 mm, there was a significant difference in the impact fracture energy when the specimen-supporting jig had an inner diameter of 6 mm, 7 mm, and 11 mm, while it was almost identical impact fracture energy when the inner diameter was 8‒10 mm. There was no significant difference in the fracture energy among 8‒10-mm inner diameters of the jig when the striker diameter was 6‒8 mm. Accordingly, when the specimen thickness was 1 mm, an equivalent impact fracture energy was acquired at 6‒10 mm diameter by setting the inner diameter of the specimen-supporting jig at 8‒10 mm. 

Regarding the 1.5 mm-thick specimens, the fewest combinations showed a significant difference (Table 1, Table 2 and Table 3). When the inner diameter of the specimen-supporting jig was 11 mm, a significant difference was noted for many combinations. When the striker diameter was 10 mm, the 6 mm inner diameter of the specimen-supporting jig was too small, which may explain why the impact fracture energy was smaller than those at other inner diameters. Accordingly, at 6‒10-mm striker diameter, an impact fracture energy not dependent on the test condition may be acquired by setting the inner diameter of the specimen-supporting jig at 7‒10 mm. 

For the 2 mm-thick specimens, when the striker diameter was 6 mm, a slightly elevated impact fracture energy was noted when the inner diameter of the specimen-supporting jig was 6 mm. This slightly higher impact fracture energy was also noted when the specimen-supporting jig had inner diameters of 6 mm and 7 mm, when the striker diameter was 8 or 10 mm. This phenomenon was not observed in specimens of other thicknesses, indicating that, when the specimen strength is high, the impact energy of the striker is directly absorbed by the jig when its inner diameter is too small relative to the striker diameter, resulting in an apparently high measured value. Therefore, when the striker diameter was 6 mm, the impact fracture energy became almost equal to that of a specimen-supporting jig with an inner diameter of 7 mm or larger, suggesting that an almost equal impact fracture energy can be acquired even at an inner diameter of 11 mm, because the specimen is strong. When the striker diameter was 8 or 10 mm, the impact fracture energy became equivalent for specimen-supporting jigs of 8‒11 mm. Accordingly, with 2 mm-thick specimens, an impact fracture energy not dependent on the test condition may be acquired by using any striker if the inner diameter of the specimen-supporting jig is 8‒11 mm. 

The E_50%_ represents the energy producing an impact fracture in 50% of specimens, based on the results of 20 specimens, following ISO specifications. The E_50%_ acquired was consistent with the median value of the impact fracture energy under the corresponding experimental condition, with a small error percentage, as shown in Table 4. Although the E_50%_ is determined by the ISO specifications, it is not appropriate for investigating the experimental conditions, because information on data distribution, such as the mean and standard deviation, median, or inter-quartile range of the impact fracture energy, cannot be acquired, suggesting that the use of impact fracture energy is superior for assessing an impact fracture testing method. 

The impact fracture energy of the acrylic resin used in this study, as reported by the manufacturer, was 18 kJ/m^2^ in terms of unnotched Charpy impact strength and 1.8 kJ/m^2^ for notched Izod impact strength [25]. The impact strengths calculated using the energy (J) obtained and the cross-sectional area of the specimens were 0.74‒1.15 kJ/mm^2^ in the 1.0 mm specimens, 1.12‒1.37 kJ/mm^2^ in the 1.5 mm specimens, and 1.88‒2.46 kJ/mm^2^ in the 2.0 mm specimens. In a previous report that compared the impact strength measured by three types of impact tests (Charpy, Izod, and weight-drop tests), it was concluded that the impact strength determined by the weight-drop test was smaller than that determined by the other two methods [26]. In our study, the impact strength of the 2.0 mm-thick specimens measured by the weight-drop impact test was larger than that determined by the notched Izod impact test reported by the manufacturer, suggesting that a specimen thickness of 2.0 mm is too large for this test condition. 

Federlin et al. [27] reported that a 1.5‒2.0 mm thickness is necessary for stress-bearing areas of ceramics restoration. Considering the application of the weight-drop impact test on materials used for crown restorations, such as ceramics and resin composites, a thickness of 1.0 mm should be excluded from the testing condition to ensure the clinical significance of the test. The impact fracture energy, E (J), acquired from the test results, should be analyzed according to the specimen thickness. 

In conclusion, based on the findings of our study, the impact resistance of acrylic resin, which can obtain only small-sized specimens, can be tested using our prototype weight-drop impact testing machine on 12 mm diameter disc-shaped specimens under test conditions in which the specimen thickness is 1.5 mm, the striker diameter is 6‒10 mm, and the inner diameter of the specimen-supporting jig is 8‒10 mm.

## Figures and Tables

**Table 1:** Multiple comparison of impact fracture energy on 1 mm thickness. *: significantly different (*p* < 0.05), **: highly significantly different (*p* < 0.01).

		St-6						St-8						St-10					
		6	7	8	9	10	11	6	7	8	9	10	11	6	7	8	9	10	11
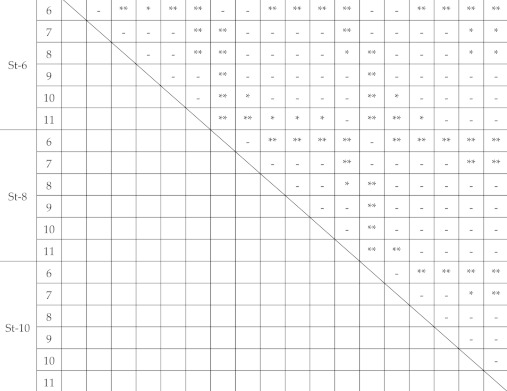																			

**Table 2:** Multiple comparison of impact fracture energy on 1.5 mm thickness. *: significantly different (p<0.05), **: highly significantly different (p<0.01).

		St-6						St-8						St-10					
		6	7	8	9	10	11	6	7	8	9	10	11	6	7	8	9	10	11
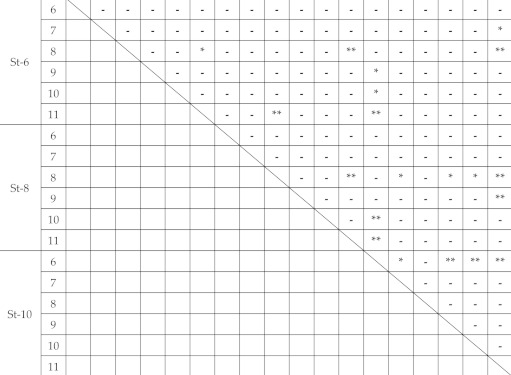																			

**Table 3:** Multiple comparison of impact fracture energy on 2 mm thickness. *: significantly different (*p* < 0.05), **: highly significantly different (*p* < 0.01).

		St-6						St-8						St-10					
		6	7	8	9	10	11	6	7	8	9	10	11	6	7	8	9	10	11
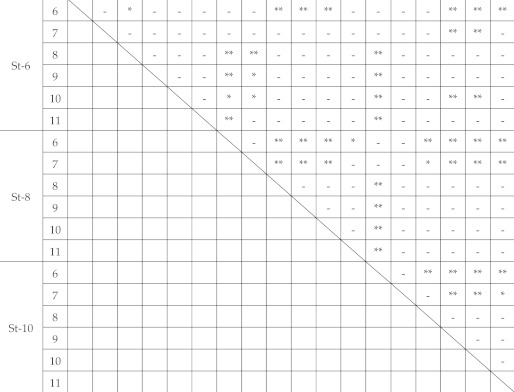																			

**Table 4:** E_50%_ (J) and the median (J) of impact fracture energy under each experimental condition.

		Diameter of the Striker					
		6 mm		8 mm		10 mm	
Thickness	I.D.	E_50%_	median	E_50%_	median	E_50%_	median
1 mm	6	0.0092	0.00884	0.0083	0.00885	0.0090	0.00984
	7	0.0108	0.0108	0.0097	0.00984	0.0102	0.01032
	8	0.0113	0.0118	0.0114	0.0118	0.0116	0.0118
	9	0.0115	0.0128	0.0117	0.0118	0.0119	0.0128
	10	0.0120	0.0128	0.0115	0.0123	0.0125	0.0128
	11	0.0132	0.0138	0.0132	0.0138	0.0131	0.0138
1.5 mm	6	0.0215	0.02015	0.0241	0.0236	0.0190	0.0202
	7	0.0209	0.0211	0.0216	0.0226	0.0233	0.0241
	8	0.0205	0.0206	0.0199	0.0207	0.0216	0.0216
	9	0.0219	0.0226	0.0210	0.0207	0.0222	0.0226
	10	0.0220	0.0221	0.0220	0.0226	0.0224	0.0226
	11	0.0230	0.0236	0.0228	0.0236	0.0237	0.0246
2 mm	6	0.0525	0.0531	0.0555	0.059	0.0558	0.0589
	7	0.0509	0.0511	0.0547	0.059	0.0532	0.0569
	8	0.0473	0.0492	0.0460	0.0472	0.0487	0.0471
	9	0.0486	0.0492	0.0460	0.0472	0.0447	0.0451
	10	0.0470	0.0511	0.0461	0.0452	0.0448	0.0451
	11	0.0491	0.0492	0.0487	0.0492	0.0462	0.0481
